# Atypical Presentations of Epiploic Appendagitis: Early Diagnosis and Non-Operative Management is the Optimal Therapy

**DOI:** 10.4021/gr422w

**Published:** 2012-03-20

**Authors:** Zackariah Clement

**Affiliations:** The Canberra Hospital, Canberra, Australia. Email: zackariahc@yahoo.com

**Keywords:** Epiploic appendagitis, Abdominal pain, Flank pain, Groin pain

## Abstract

Epiploic appendagitis is a benign self-limiting inflammation of the appendices epiplocae of the colon. It often presents as abdominal pain rarely accompanied by fever, nausea, or vomiting or any other abdominal symptoms. It can mimick acute diverticulitis or appendicitis on clinical exam. The diagnosis of epiploic appendagitis primarily relies on imaging modalities such as CT. The treatment is analgesia and non-steroidal anti-inflammatory drugs are usually sufficient to control pain and no surgical intervention is needed. This report illustrates two cases with epiploic appendagitis who presented with atypical symptoms, clinical and radiological findings and management of this condition.

## Introduction

Epiploic appendagitis is an inflammation of the appendices epiploicae of the colon. It is also known as appendicitis epiploicae, or appendagitis [1-4]. It is considered to be a benign and self-limiting condition of the epiploic appendages that affects middle-aged men, although a wide range has been reported [2, 3]. It often presents as abdominal pain rarely accompanied by fever, nausea, or vomiting or any other abdominal symptoms. It is often misdiagnosed due to the lack of pathognomonic clinical features and also because it is a rare condition and the more common diagnoses of abdominal pain such as diverticulitis and appendicitis are considered [2-5].

This report illustrates two cases with epiploic appendagitis who presented with atypical symptoms, clinical and radiological findings and management of this condition.

## Case Report

### Case 1

A 58 years old male with a background of hypertension presented to the emergency department with 3 days history of worsening low back and left flank pain. It came on suddenly and was sharp stabbing, non-radiating, non-migratory pain associated with nausea and decreased appetite. He had no urinary symptoms and no change in bowel habits. On physical examination he was overweight with central obesity (BMI of 26) and appeared uncomfortable and grimacing in pain. He was afebrile and haemodynamically stable. His abdomen showed signs of localised peritonism in the left iliac fossa with voluntary guarding and percussion tenderness. He complained of dull ache around the left lower lumbar area over the erector spinae, but no muscle or bony tenderness. He was neurologically intact.

Blood tests showed mildly elevated neutrophil of 8 x 10^9^/L and raised inflammatory markers with a C-reactive protein (CRP) of 54 mg/L. Liver and renal function tests were within normal limits. Chest and abdominal radiograph were performed, which did not show any evidence of infection, pneumoperitoneum, or bowel obstruction.

He was initially diagnosed with acute diverticulitis in the emergency department and treated with intravenous antibiotics. Further radiological investigation was performed with a computed tomography (CT) scan of the abdomen and pelvis with and without contrast showed inflammatory changes in the left iliac fossa anteriorly with mesenteric stranding surrounding an area of fat density. These changes were anterior to the distal descending colon. There was no bowel wall thickening or features of obstruction. No significant diverticulosis was demonstrated. These changes in the left iliac fossa were consistent with epiploic appendagitis. He was admitted for overnight observation and treated non-operatively with analgesia, intravenous fluids and bowel rest. He was discharged home with an oral analgesia and anti-inflammatory agent.

### Case 2

A 43 year old male presented to the emergency department with sudden onset of severe left groin pain, which was not associated with fever, nausea, vomiting, or diarrhoea. His past medical history include depression and being treated with Escitalopram. He had no bladder or bowel symptoms. On physical examination, the patient was noted to be in distress, diaphoretic, supporting his abdomen with a pillow. Haemodynamically he was in sinus tachycardia with a pulse rate of 112 beats per minute, but not febrile. The abdomen was tender in the left lower quadrant, particularly over the inguinal ligament area, with voluntary guarding but no rebound or percussion tenderness. No inguinal hernia was found.

Blood test showed elevated CRP of 88 mg/L. Full blood count and liver function tests were within normal limits. Abdominal radiograph was reported normal. He was initially diagnosed with acute diverticulitis in emergency department and treated with intravenous antibiotics. Further imaging with a computer tomography (CT) scan of abdomen and pelvis showed ill-defined mesenteric fat stranding of the mesenteric fat adjacent to the descending and sigmoid colon surrounding an area of fat density, findings consistent with epiploic appendagitis. Antibiotics were stopped and he was treated non-operatively with analgesia, non-steroidal anti-inflammatory and intravenous fluids. He was admitted and observed overnight with good improvement and discharged the following morning on oral analgesia.

## Discussion

Epiploic appendages are small out-pouchings of fat-filled, serosa-covered structures, situated along the entire colon and are more abundant and larger in the transverse and sigmoid colon [3]. Each appendage is supplied by one or two small colonic end-arteries and a small draining vein. For reasons not known, they are largest and most prominent in obese persons and in those who have recently lost weight [4, 6, 7]. They are presumed to serve a protective and defensive mechanism similar to that offered by the greater omentum. They may represent a site of fat storage to be accessed in prolonged periods of starvation and also act as a protective cushion during peristalsis [2, 3].

Epiploic appendagitis is usually caused by torsion, but the reason remains unclear [3-8]. The vein, which is longer than the artery by virtue of its tortuous course, alters the anatomy such that the pedicle is predisposed to twisting. This can cause venous thrombosis of a draining vein and can also predispose to twisting of the appendage pedicle and lead to aseptic fat necrosis [3, 6, 7]. Epiploic appendagitis may occur anywhere in the colon; however, the surgical literature suggests that 57% of cases occur in the rectosigmoid junction, 26% in the ileocecal region, 9% in the ascending colon, 6% in the transverse colon and 2% in the descending colon and usually occurs in males in the fourth and fifth decades [8-10]. Depending on its location, it can mimic many disorders causing an acute abdomen such as colonic diverticulitis, acute appendicitis, a gynaecological disorder or acute cholecystitis. The diagnosis is difficult to make clinically due to the non-specific signs and symptoms exhibited by this condition. Most patients describe the pain as dull, constant and non-radiating, non-migrating, and physical examination reveals localised tenderness [1, 11, 12]. The results of laboratory tests also seem to be non-specific. Abnormal laboratory parameters may include slightly elevated serum levels of C-reactive protein and neutrophil leucocytes. However, all routine laboratory parameters, such as erythrocyte sedimentation rate, liver and pancreatic enzymes, are usually within normal limits [2, 3, 8, 12]. The diagnosis of epiploic appendagitis primarily relies on imaging modalities and is made most often on CT [6, 7, 9]. In normal conditions, epiploic appendages are not detectable on a CT scan unless surrounded by an intra-peritoneal fluid such as ascites or haemoperitoneum [12-14]. Generally, the epiploic appendages have fat attenuation and resemble other adipose structures unless they are inflamed. The most common findings on CT are inflammatory changes with mesenteric stranding, fat-density oval lesion with surrounding inflammation on the anterior aspect of the sigmoid colon [4, 6, 7, 9, 13]. A diagnosis of epiploic appendagitis should always be considered as part of the differential diagnosis of abdominal pain due to its self-limiting behaviour. The treatment is non-operative with analgesia and non-steroidal anti-inflammatory drugs are usually sufficient to control pain and no surgical intervention is needed [1-5, 12]. Non-operative management reduces complications from general anaesthetic and surgical intervention. It also reduces the costs incurred and time spent managing this condition [2-4].

With non-operative management, most patients' symptoms are alleviated between a few days and 4 weeks. Careful clinical examination and appropriate diagnosis of epiploic appendagitis with imaging procedures, specifically CT, enables successful non-operative, outpatient treatment of patients with epiploic appendagitis. Such an approach avoids unnecessary abdominal surgery and associated additional health-care costs.
